# Perioperative outcomes of neonatal versus delayed surgery for Hirschsprung disease: a nationwide retrospective cohort study in Japan

**DOI:** 10.1007/s00383-025-06126-3

**Published:** 2025-07-14

**Authors:** Mai Kutsukake, Shotaro Aso, Takaaki Konishi, Michimasa Fujiogi, Naohiro Takamoto, Yoshitsugu Yanagida, Kaori Morita, Kiyohide Fushimi, Hiroki Matsui, Hideo Yasunaga, Jun Fujishiro

**Affiliations:** 1 Department of Pediatric Surgery, Graduate School of Medicine, The University of Tokyo, 7-3-1 Hongo, Bunkyo-Ku, Tokyo Japan; 2 Department of Clinical Epidemiology and Health Economics, School of Public Health, The University of Tokyo, 7-3-1 Hongo, Bunkyo-Ku, Tokyo Japan; 3 Department of Health Service Research, School of Public Health, The University of Tokyo, 7-3-1 Hongo, Bunkyo-Ku, Tokyo Japan; 4 Department of Health Policy and Informatics, Graduate School of Medical and Dental Sciences, Institute of Science Tokyo, 1-5-45 Yushima, Bunkyo-Ku, Tokyo Japan

**Keywords:** Morbidity, Length of stay, Costs and cost analysis

## Abstract

**Purpose:**

To assess the safety and short-term outcomes of neonatal surgery for Hirschsprung disease.

**Methods:**

This retrospective cohort study extracted data from a nationwide Japanese inpatient database from July 2010 to March 2021. Patients diagnosed with Hirschsprung disease within 30 days of life who underwent definitive surgery within 120 days of life were identified. Patients who underwent enterostomy before definitive surgery and those who were diagnosed with the extended type of Hirschsprung disease were excluded. We stratified patients who underwent definitive surgery within 30 days of life into the neonatal group (n = 65), and the others into the non-neonatal group (n = 300). Propensity-score overlap weighting analyses were employed to compare the outcomes between the two groups.

**Results:**

Overlap weighting analysis revealed no significant difference in in-hospital morbidity (risk difference [95% confidence interval], −4.6 [−16.8–7.5]). Although the post-operative length of stay was longer (difference, 6.5 [−0.1 to −13.0] days), the total length of stay during infancy (difference, 14.6 [6.3 to 22.8] days) was shorter in the neonatal group than in the non-neonatal group.

**Conclusions:**

This nationwide cohort study found that although in-hospital morbidity was similar between definitive surgery for Hirschsprung disease performed in the neonatal and non-neonatal periods, neonatal surgery was associated with a shorter total length of hospitalization during infancy compared with delayed surgery. In terms of the short-term outcomes, neonatal surgery for Hirschsprung disease is safe, and delayed surgery may not be beneficial.

**Supplementary Information:**

The online version contains supplementary material available at 10.1007/s00383-025-06126-3.

## Introduction

Hirschsprung disease is a congenital intestinal malformation characterized by the absence of ganglionic cells in the submucosal and myenteric plexuses of the distal bowel, with a prevalence of one in 5000 live births [1]. Resection of the aganglionic segment and anastomosis of the residual intestine to the anus constitute definitive surgery for Hirschsprung disease.

Staged definitive surgery preceded by enterostomy was originally recommended beyond the age of 4 months [2]. Recently, primary definitive surgery is likely to be performed at a young age because the diagnosis can be confirmed in the neonatal period [3], and surgical and anesthetic techniques for infants have improved. The median age at definitive surgery in the United States was 6 months in 1997 and 1 month in 2006 [4].

However, the safety and advantages of definitive primary surgery in the neonatal period remain unclear. A previous study reported that definitive surgery in neonates was safe, because the incidence of perioperative complications and postoperative enterocolitis did not differ significantly between neonates and non-neonates [5]. Meanwhile, other studies have demonstrated worse short-term outcomes, an elevated risk of perioperative complications and postoperative enterocolitis [6], longer postoperative length of stay, and longer total hospital stay after surgery in the neonatal compared with the non-neonatal period [7]. Additionally, none of these studies adjusted for patient background when comparing neonates and non-neonates.

Therefore, this study aimed to compare the in-hospital morbidity, length of hospital stay, and hospitalization costs of primary definitive surgery for Hirschsprung disease in the neonatal versus non-neonatal periods, using a nationwide inpatient database in Japan.

### Methods

#### Data source

This nationwide retrospective cohort study was conducted using the Diagnosis Procedure Combination database, which includes hospital administrative claims and discharge abstract data recorded by attending physicians during hospitalization [8]. Participation of university hospitals in the database is mandatory, while that of community hospitals is voluntary. The database covers 88% of hospitals with neonatal intensive care units [9]. The requirement for informed consent was waived due to the anonymous nature of the data. This study adhered to the STROBE guidelines [10] and was approved by the Institutional Review Board of the University of Tokyo (approval number: 3501-(5); May 19, 2021).

The database includes the following data: unique hospital identifier, sex, age, birth weight, gestational age, primary diagnoses and comorbidities on admission, complications after admission recorded in the International Classification of Diseases, Tenth Revision (ICD-10) codes, original Japanese procedural codes, drugs used, length of stay, and discharge status. This database possesses good sensitivity and specificity for diagnosis and procedural records [11, 12].

#### Study protocol

We retrospectively identified patients who were hospitalized with a diagnosis of Hirschsprung disease (ICD-10 code: Q431) within 30 days of birth and underwent primary definitive surgery within 120 days of birth between July 2010 and March 2021. Definitive surgery for Hirschsprung disease was identified using the Japanese procedural codes. Patients who underwent enterostomy before definitive surgery (i.e., staged surgery) were excluded. Patients diagnosed with the extended type of Hirschsprung disease (identified by the Japanese text code) or allied disorders of Hirschsprung disease (ICD-10 code: Q432) were also excluded. Eligible patients were divided into two groups: those who underwent definitive surgery within 30 days of birth (neonatal group) and those who underwent definitive surgery between 31 and 120 days of birth (non-neonatal group). The cutoff period used to define the groups was chosen according to a previous study [13].

We described the baseline characteristics of both groups, including the patient’s sex, gestational age, birth weight, congenital anomalies, type of hospital (academic or non-academic), hospital volume, preoperative intensive care unit stay within 30 days of birth, fiscal year at surgery, and surgical procedure (laparoscopic or non-laparoscopic). We categorized gestational age into <37 and ≥37 weeks, and birth weight into <2500 and ≥2500 g. Congenital anomalies were defined using ICD-10 codes (Supplementary Table 1). Hospital volume was categorized into two groups, with approximately equal numbers of patients allocated to each group, according to the annual number of definitive surgeries for Hirschsprung disease at each hospital. We chose these covariates based on a previous study [14].

The primary outcome was in-hospital morbidity, which was defined as a composite outcome of diagnoses of bleeding, surgical-site infection, sepsis, enterocolitis, bowel obstruction, bowel ischemia and perforation, peritonitis, anal or intestinal stricture, sphincter malfunction, anal prolapse, fistula formation, incisional hernia, and general morbidity (urinary tract infection and respiratory complications) during hospitalization for definitive surgery [14]. We also designated the requirement for additional surgical procedures (e.g., redo surgery and surgeries for complications) as morbidity. Details of the diagnoses according to the ICD-10 codes and Japanese procedural codes are presented in Supplementary Table 1.

The secondary outcomes included duration of anesthesia on the day of definitive surgery, blood transfusion, reoperation, postoperative length of stay, prescription for bowel management (enema and laxative) at discharge, cost of hospitalization for definitive surgery, readmission within 30 and 365 days after discharge, and pre- and post-operative enterocolitis during the entire study period. We also assessed the total length of hospital stay and hospitalization costs in the first year of life. The cost was calculated using a currency exchange rate of JPY 140 per USD.

#### Statistical analysis

To compare the outcomes between the two groups, we conducted propensity-score overlap weighting analysis to balance the covariates between the two groups and mimic a randomized trial without excluding participants from the study sample [15, 16]. Propensity scores were calculated using a logistic regression model where the dependent variable was the receipt of surgery within 30 days of birth, and the background characteristics were designated as the independent variables. Overlap weighting was conducted by weighting patients according to the predicted probability of receiving the opposite treatment [17]. Thus, patients in the neonatal group were weighted by the probability of receiving surgery after 30 days of life (1-propensity score), whereas those in the non-neonatal group were weighted by the probability of receiving surgery within 30 days (propensity score). After overlap weighting, a <10% absolute standardized difference in the covariates indicated a negligible difference [18]. The risk difference between the neonatal and non-neonatal groups was calculated. Multiple imputations were employed to replace each missing value with a set of substituted plausible values by creating 20 filled-incomplete datasets using a Markov chain Monte Carlo algorithm known as “chained equations imputation” because some values for gestational age and birth weight were missing [19]. Multiple imputation assumes that data are missing at random, and that any systematic differences between the missing and observed values can be explained by differences in the observed data [20, 21].

For summary statistics, we performed the chi-squared test for binary and categorical variables and the t-test for continuous outcomes. All 95% confidence intervals and P-values were calculated on two-sided hypothesis tests, where *P* < 0.05 denotes statistical significance. All statistical analyses were conducted using Stata/SE 18.0 (StataCorp, College Station, TX, USA).

## Results

We identified 928 patients diagnosed with Hirschsprung disease during hospitalization within 30 days of birth. Of them, 396 underwent definitive surgery for Hirschsprung disease within 120 days of birth. We excluded 20 patients who underwent prior enterostomy, eight patients who were diagnosed with long-segment Hirschsprung disease, and three patients diagnosed with allied disorders of Hirschsprung disease. Finally, 365 patients were eligible for this study, of whom 65 underwent definitive surgery for Hirschsprung disease in the first 30 days of life and 300 patients underwent definitive surgery within 31–120 days.

Table 1 summarizes the baseline characteristics of all patients and propensity-score overlap-weighted patients. The neonatal group had fewer congenital anomalies, a higher proportion of treatment at academic hospitals and high-volume hospitals, and higher preoperative intensive care unit admissions compared to the non-neonatal group. Although some fluctuations were found in the year of surgery, no overall trends were detected.

**Table 1:** Background characteristics of all patients and propensity-score overlap-weighted patients who underwent definitive surgery for Hirschsprung disease during the neonatal period and later (non-neonatal*)

	Crude analysis					Overlap weighting analysis†		
	Neonatal		Non-neonatal			Neonatal	Non-neonatal	
	n = 65 65		n = 300		ASD (%)			ASD (%)
**Male sex**	54	(83.1)	245	(81.7)	2.5	(82.4)	(82.4)	0.0
**Gestational age category, week**								
<37	2	(3.1)	12	(4.0)	14.0	(4.4)	(4.4)	0.0
≥37	54	(83.1)	223	(74.3)		(95.6)	(95.6)	0.0
missing	9	(13.9)	65	(21.7)				
**Birth weight category, g**					22.1			
>2500	2	(3.1)	12	(4.0)		(4.4)	(4.4)	0.0
≤2500	54	(83.1)	223	(74.3)		(95.6)	(95.6)	0.0
missing	9	(13.9)	65	(21.7)				
**Congenital anomalies**	9	(13.9)	53	(17.7)	17.3	(13.7)	(13.7)	0.0
**Academic hospital**	59	(90.8)	171	(57.0)	90.2	(88.0)	(88.0)	0.0
**Hospital volume, per year**								
<2.27	26	(40.0)	158	(52.7)	18.9	(45.4)	(45.4)	0.0
≥2.27	39	(60.0)	142	(47.3)		(54.6)	(54.6)	0.0
**ICU stay before surgery within 30 days of life**	45	(69.2)	159	(53.0)	24.4	(70.6)	(70.6)	0.0
**Year of surgery**								
2010	9	(13.9)	7	(2.3)	38.2	(6.6)	(6.6)	0.0
2011	7	(10.7)	24	(8.0)	12.9	(13.2)	(13.2)	0.0
2012	7	(10.7)	25	(8.3)	6.7	(9.1)	(9.1)	0.0
2013	2	(3.1)	32	(10.7)	27.6	(4.3)	(4.3)	0.0
2014	6	(9.2)	31	(10.3)	2.6	(9.2)	(9.2)	0.0
2015	5	(7.7)	22	(7.3)	4.6	(8.5)	(8.5)	0.0
2016	4	(6.2)	29	(9.7)	4.1	(7.3)	(7.3)	0.0
2017	6	(9.2)	30	(10.0)	4.8	(10.3)	(10.3)	0.0
2018	6	(9.2)	33	(11.0)	15.4	(9.7)	(9.7)	0.0
2019	6	(9.2)	26	(8.7)	15.5	(9.5)	(9.5)	0.0
2020	3	(4.6)	21	(7.0)	7.4	(5.6)	(5.6)	0.0
2021	4	(6.2)	20	(6.7)	2.1	(6.6)	(6.6)	0.0

ASD, absolute standard difference; ICU, intensive care unit.

*Patients in the neonatal group underwent surgery within 30 days of life, while those in the non-neonatal group underwent surgery within 31–120 days.

†One of the imputed datasets. Data are presented as n (%).

The crude outcomes of all the patients are shown in Table 2. The neonatal group had a longer postoperative length of stay (21 vs. 14 days) and bore higher hospitalization costs for definitive surgery (37,812 vs. 17,312 USD) compared to the non-neonatal group. The duration of anesthesia (338 vs. 354 min), in-hospital morbidity (20.0% vs. 15.0%), and hospitalization costs during the first year of life (38,406 vs. 29,839 USD) did not differ between the two groups. Redo surgeries were performed on days 16 and 50 after the first definitive surgery for two patients in the neonatal group, and on day 35 for one patient in the non-neonatal group. Corrective operations for complications were conducted on days 3–21 after definitive surgery for three patients in the neonatal group, and on days 2–19 for five patients in the non-neonatal group.

**Table 2:** Comparison of outcomes of definitive surgery for Hirschsprung disease between the neonatal and later periods (non-neonatal) in the crude analysis

	Neonatal		Non-neonatal		P-value
	n = 65 65		n = 300 308		
In-hospital morbidity	12	(18)	41	(14)	0.32
Reoperation during hospitalization	4	(6.2)	6	(2.0)	0.063
Redo surgery	2	(3.1)	1	(0.33)	0.026
Operation for complications	3	(4.6)	5	(1.7)	0.14
Blood transfusion during hospitalization	5	(7.7)	33	(11)	0.43
Bowel management prescription at discharge	20	(31)	88	(29)	0.82
Enema	18	(28)	72	(24)	0.53
Laxative	2	(3.1)	11	(3.7)	0.82
Readmission within 30 days	7	(11)	41	(14)	0.53
Readmission within 365 days	18	(28)	85	(28)	0.92
Pre-operative enterocolitis	6	(9.2)	22	(7.3)	0.60
Post-operative enterocolitis	12	(19)	77	(26)	0.22

	Median	IQR	Median	IQR	P-value
Duration of anesthesia for definitive surgery, min	338	269–445	354	292–423	0.35
Postoperative length of stay, days	21	16–26	14	10–20	0.003
Total length of stay during the 1st year of life, days	44	38–52	50	35–71	0.98
Cost of hospitalization for definitive surgery (USD)	37,812	26,789–44,984	17,312	14,644–31,179	< 0.001
Total hospitalization cost during the 1st year of life (USD)	38,406	28,377–45,082	29,839	23,098–40,672	0.026

IQR, interquartile range.

After propensity-score overlap weighting, the baseline characteristics were balanced between the two groups (Table 1). Table 3 presents the results of the overlap weighted analysis. Compared with the non-neonatal group, the neonatal group showed a significant association with a longer postoperative length of stay (difference [95% confidence interval], −6.5 [−0.1 to −13.0] days) and higher cost of hospitalization for definitive surgery (USD −10,642.7 [−5884 to −15,345]). The duration of anesthesia (1.9 [−31.2 to 35.0] min) and in-hospital morbidity (risk difference, −4.6 [−16.8 to 7.5]) did not differ between the two groups. Regarding the outcomes during the first year of life, the total length of stay in the neonatal group was significantly shorter than that in the non-neonatal group (difference, 14.6 [6.3 to 22.8] days), whereas the total hospitalization cost did not differ significantly between the two groups (USD −1868 [−6925 to 3,187]).

**Table 3:** Comparison of outcomes of definitive surgery for Hirschsprung disease between the neonatal and later (non-neonatal) periods in the propensity-score overlap weighting analysis

	Neonatal	Non-neonatal	Risk difference	95% CI	P-value
In-hospital morbidity	(19)	(14)	−4.6	−16.8 to 7.5	0.47
Reoperation during hospitalization	(6.2)	(2.2)	−4.0	−0.5 to 2.5	0.23
Redo surgery	(3.8)	(0.5)	−3.0	−7.9 to 2.2	0.27
Operation for complications	(4.4)	(1.6)	−2.3	−8.4 to 2.5	0.29
Blood transfusion during hospitalization	(6.6)	(14)	7.0	1.5 to 15.6	0.11
Bowel management prescription at discharge	(31)	(30)	−0.2	− 13.9 to 13.6	0.98
Enema	(27)	(27)	0.4	−12.8 to 13.7	0.95
Laxative	(3.8)	(4.4)	0.7	−5.4 to 6.8	0.83
Readmission within 30 days	(9.2)	(14)	5.0	−3.7 to 13.6	0.27
Readmission within 365 days	(26)	(30)	3.8	−9.3 to 16.8	0.58
Preoperative enterocolitis	(7.7)	(7.5)	0.0	7.1 to 7.1	0.99
Postoperative enterocolitis	(21)	(27)	6.5	−6.3 to 19.3	0.32

	Median	IQR	Median	IQR	Difference	95% CI	P-value
Duration of anesthesia for definitive surgery, min	339	270–439	354	295–431	1.9	−31.2 to 35.0	0.92
Postoperative length of stay, days	21	15–26	14	10–21	−6.5	−0.1 to −13.0	0.048
Total length of stay during the 1st year of life, days	44	38–52	55	40–76	14.6	6.3 to 22.8	<0.001
Cost of hospitalization for definitive surgery (USD)	38,406	27,566–45,082	20,221	14,727–37,353	−10,615	−5,884 to −15,346	<0.001
Total hospitalization cost during 1st year of life (USD)	39,464	28,760–45,099	34,442	27,063–42,579	−1868	−6,925 to 3,188	0.48

IQR, interquartile range.

## Discussion

In the current study, we investigated the short-term outcomes of patients who were diagnosed with Hirschsprung disease within 30 days of birth and underwent definitive surgery within 120 days without preceding enterostomy, using data extracted from a nationwide Japanese database. This is the first study to balance the patients’ background characteristics for comparison between neonatal and non-neonatal surgery for Hirschsprung disease and to mimic a randomized trial, utilizing the power of a large-scale database. Overlap weighted analysis revealed no significant differences in in-hospital morbidity and hospital costs during the first year of life between the two groups. However, definitive surgery within 30 days of birth was associated with a shorter total length of stay during the first year of life compared to non-neonatal definitive surgery.

Previous studies investigating the short-term complications of definitive surgery in the neonatal period have yielded discrepant results. One study reported a higher incidence of complications such as anastomotic stricture and leakage in primary definitive surgery for neonates than that in non-neonates [6] while another study found no significant difference in perioperative complications between primary definitive surgery for neonates and non-neonates [5]. Moreover, neither of these studies adjusted for patient background. In the present study, we balanced background covariates between the neonatal and non-neonatal groups and mimicked a randomized trial using propensity-score overlap weighting analysis. This analysis found no significant difference in in-hospital morbidity between the two groups, albeit a shorter cumulative hospital stay during infancy was evident in the neonatal group. These results would support early definitive surgery in neonates.

Delaying definitive surgery even for a short time may have some pathological drawbacks because definitive surgery performed immediately in the neonatal period is associated with fewer unplanned readmissions, reduced gastrointestinal complications on readmission, and lower hospitalization costs for readmissions compared to delayed primary definitive surgery in the neonatal period [22]. Delaying definitive surgery without a diverting enterostomy and maintaining the aganglionic region may have a deleterious effect on the patient.

However, the pathological effects of the delay in surgery on patients are unknown. Previous studies have posited that potential pathology of intestinal damage in Hirschsprung disease includes alterations in gut microbiota homeostasis and immunity [23, 24], and inflammatory changes caused by the chronic accumulation of stool. Histologically, inflammation caused by the chronic accumulation of stool may result in thickening of the muscular layers, mucosal ulceration, and fibrous adhesions between the mucosa and submucosa [25]. This preoperative damage occurring in the segment of the intestine proximal to the aganglionic region could remain for a considerable time even after definitive surgery because previous studies demonstrated that preoperative morbidity (enterocolitis) was related to postoperative enterocolitis [26, 27], and half of postoperative enterocolitis occurred more than 1 year after definitive surgery [28].

The findings pertaining to postoperative length of stay, in-hospital morbidity, and readmission in our study were similar to those of a previous study [13]. Although the in-hospital morbidity was similar between the groups, the post-operative length of stay was longer in the neonatal group than in the non-neonatal group. Physicians may have been cautious about increasing the amount of milk or deciding the timing of discharge for young patients, even if the post-operative course was uneventful. We found no significant differences in hospitalization costs during infancy between the neonatal and non-neonatal groups. Moreover, the cumulative length of hospital stay (days) during the first year of life was shorter in the neonatal group than in the non-neonatal group. Two possible reasons exist for this observation. First, guidance for home care, such as enema and irrigation, may have required more time and prolonged the initial hospitalization in the non-neonatal group. Prolonged hospitalization can result in higher medical costs. Second, the non-neonatal group may have incurred higher readmission costs due to gastrointestinal complications. A previous study comparing neonatal definitive surgery with delayed definitive surgery with or without a preceding enterostomy reported higher readmission costs due to gastrointestinal complications [22].

The present study had some limitations. First, the database lacked clinical information on the severity of Hirschsprung disease, such as symptoms and length of the aganglionic lesion. We believe that the disease severity in this study population was limited because the study population was confined to those diagnosed during the neonatal period and could undergo definitive surgery without preceding enterostomy. Second, data on the surgical technique used were not available. Third, a substantial number of values for gestational age and weight were missing. We performed multiple imputation to minimize selection bias arising from these missing values. Fourth, data on the long-term fecal control outcomes were unavailable. However, several longitudinal studies have demonstrated that fecal control of patients with Hirschsprung disease improved with age and showed no significant difference in the long term with respect to the age at definitive surgery or upon comparison with healthy controls [26, 29, 30]. Fifth, readmissions were traceable only when patients were admitted to the same facility. Sixth, although the database possesses good sensitivity and specificity for the diagnosis and procedural records of several diseases [11, 12], no study has validated the recorded diagnoses of the in-hospital morbidities investigated in the present study, and these outcomes may have been underestimated.

In conclusion, among patients diagnosed with Hirschsprung disease within 30 days of birth, definitive surgery within 30 days of birth did not increase in-hospital morbidity and was associated with a shorter length of hospital stay during infancy than that of definitive surgery performed within 31–120 days of birth. Although the present study focused on short-term outcomes and did not verify the long-term fecal outcomes, delaying definitive surgery for Hirschsprung disease may not be beneficial to patients in terms of the length of hospital stay during infancy.

## Supplementary Information

Below is the link to the electronic supplementary material.

Supplementary file1 (DOCX 19 KB)

## Declarations

### Data availability

No datasets were generated or analysed during the current study.

### Data statement

The datasets analyzed in this study are not publicly available because of contracts with the hospitals providing data to the database.
