# Supplementary material for: Perioperative outcomes of neonatal versus delayed surgery for Hirschsprung disease: a nationwide retrospective cohort study in Japan
Source: Pediatr Surg Int. 2025 Jul 14;41(1):211. doi: 10.1007/s00383-025-06126-3 (PMC12259758; doi:10.1007/s00383-025-06126-3)
Supplement: Supplementary file 1 — Supplementary file1 (DOCX 19 KB) [file 383_2025_6126_MOESM1_ESM.docx]

Supplementary Table.1 Codes for morbidities, complications, and interventions

|  | ICD-10 codes for morbidities and complication |
| --- | --- |
| Bleeding | K625 K661 K922 R571 R58 T810 T811 |
| Surgical-site infection | T793 T814 K610 K612 K613K614 K628 |
| Sepsis | A40 A41 |
| Enterocolitis | A00–09 K638 |
| Bowel obstruction | K560–562 K564–567 P761 P769 K913 |
| Bowel ischemia and perforation | K550 K551 K631 K632 |
| Peritonitis | K650 K658 K659 |
| Anal stricture | K624 K918* |
| Sphincter malfunction | K628 |
| Anal prolapse | K622 K623 |
| Fistula formation | K603 K604 K605 K632 T818 |
| Incisional hernia | K430–432 K420–421, K429 |
| Congenital anomalies | Q0–1 Q38–42 Q430 Q433–439 Q44–45 Q5–9 |
| Urinary tract infection | N10 N12 N30 N390 |
| Respiratory complications | J14–18 J958 J959 J960 J969 |
|  | Original Japanese codes for interventions for complications |
| Definitive surgery, Redo surgery | K735 K735-3 |
| Stoma creation and closure | K725 K725-2 K726 K727 K730 K731 K732 K736 |
| Abdominal exploratory surgery | J010 J013 K488-2 K636 K636-3 |
| Bleeding control surgery | K607 K6151 K6153 K6154 K722 |
| Infection control intervention | J003 J003-3 K630 K737 K745 |
| Bowel obstruction surgery | J034 K714 K714-2 K7151 K7152 K715-2 |
| Bowel resection and surgery for peritonitis | K636-2 K639 K639-3 K637 K637-2 K638 K640 K712 K724 K7161 K7162 K716-2 K716-21 K716-22 K7191 K7192 K7193 K719-21 K719-22 K719-5 K740 K740-2 |
| Intervention for bowel and anal stricture | J031 J032 K728 K735-2 K741 K743-2 K749 K750 K752 |
| Surgery for anal prolapse | J037 K742 K742-2 K743 K743-3 |
| Surgery for fistula | K6311 K6312 K746 K746-2 K719-4 |
| Incisional hernia operation | K6331 K633-21 K6333† K633-24† |

*Specified by Japanese text codes

†Only included in patients who underwent laparoscopic procedures
